# A Bibliometric Analysis of the Top 50 Most Cited Articles on Iatrogenic Nerve Injuries of the Upper Limb Following Surgery

**DOI:** 10.1055/a-2702-5186

**Published:** 2025-10-07

**Authors:** Saran Singh Gill, Abith Ganesh Kamath, Hussayn Shinwari, Ashley Simpson, Anna Panagiotidou, Mike Fox, Marco Sinsi, Kapil Sugand

**Affiliations:** 1 Faculty of Medicine, Imperial College London, London, United Kingdom; 2 Faculty of Medicine, St George's University of London, United Kingdom; 3 Royal National Orthopaedic Hospital, Stanmore, United Kingdom

**Keywords:** iatrogenic, upper limb, nerve, injury, surgery, most cited, bibliometric

## Abstract

**Background:**

Iatrogenic nerve injuries of the upper limb have profound impacts on patients their pain, functionality, and quality of life.

**Objectives:**

This study aims to identify and analyze the most cited publications on those iatrogenic injuries to elicit trends, thematic analysis, and reduce risk.

**Methodology:**

A bibliometric analysis was performed using the Web of Science database. Search terms included “Iatrogenic,” “Upper Limb,” “Nerve,” and “Injury.” The top 50 cited peer-reviewed publications were ranked by citation count and analyzed for publication year, journal, country of origin, institutional affiliations, authorship, and research focus. Trends in diagnostic and management practices were also evaluated.

**Results:**

The most cited articles, published between 1995 and 2022, peaked in publication frequency in 2010 and 2017 ( *n* = 4 each). Citation counts ranged from 7 to 260, with a median of 26.5 (± 15.0, 95% confidence interval: 16–75). These articles were featured in 44 journals. The United States emerged as the leading contributor in both volume and impact ( *n* = 16). Prominent themes included supracondylar humerus fractures ( *n* = 21) and humeral shaft fractures ( *n* = 10), alongside mentions of diaphyseal humeral fractures and shoulder surgery ( *n* = 4 each). Ulnar nerve injuries were the most frequently discussed ( *n* = 23), followed by injuries involving multiple nerves ( *n* = 18) and the radial nerve ( *n* = 14).

**Conclusions:**

This bibliometric analysis highlights key studies on iatrogenic upper limb nerve injuries, identifies trends and gaps, and lays a foundation for evidence-based protocols. It also serves as a guide for future research and collaborative efforts to improve prevention and treatment.

## Introduction

### Role of Bibliometric Analysis

Bibliometric analyses serve as a pivotal methodology for evaluating the credibility, quality, and influence of scholarly work, offering profound insights into research trends and academic impact. 1 2 Among the most prominent metrics employed is citation frequency, which is widely regarded as a proxy for influence within its field. 3 Publications with high citation rates often drive significant advancements and shape future research directions. Bibliometric methods, which aim to analyze and assess these articles, are therefore indispensable for identifying knowledge gaps, prioritizing funding allocation, and informing resource distribution in academic and clinical practice. 4

### Iatrogenic Upper Limb Nerve Injuries

Iatrogenic nerve trauma, a rare complication of medical treatment, accounts for 8 to 25% of all peripheral nerve injuries, with outcomes ranging from mild paraesthesia to persistent paralysis, depending on site and severity. 5 6 7 Intraoperative complications are a common cause, 8 in which injuries may result in transient or permanent sensorimotor deficits as well as intractable chronic pain. 5 These findings highlight the urgent need for enhanced preventive strategies and heightened clinical awareness to mitigate the risks of iatrogenic nerve trauma, especially since these may ultimately be avoidable in the first place.

### Need for Analyzing Iatrogenic Peripheral Nerve Injuries

Despite the growing landscape of research on peripheral nerve injuries, 9 the paucity of focused bibliometric analysis of iatrogenic upper limb nerve injuries remains as a current challenge. Given the profound functional, psychological, and socioeconomic consequences of these injuries, mapping the research landscape is essential. 10 11 Bibliometric analysis offers a systematic approach to evaluating influential studies, uncovering emerging trends, and identifying key themes.

### Aims and Objectives

By examining citation patterns and research outputs, this study aims to provide valuable insights, expose gaps in existing knowledge, and guide future investigations. These findings will ultimately inform clinical practice and policy, supporting targeted interventions to potentially reduce avoidable iatrogenic nerve injuries (INIs) to both improve clinical outcomes while upholding patient safety.

## Methodology

### Database

The Web of Science database was searched to identify all articles related to INIs of the upper limb. Web of Science integrates major databases, including Medline, Embase, the Science Citation Index Expanded, and Journal Citation Reports, offering a comprehensive platform for accessing high-quality medical and scientific literature.

### Keywords and MeSH

The keywords and MeSH (Medical Subject Headings) used for the search include terms related to the condition, anatomical regions, surgical procedures, and associated complications. These terms comprise “iatrogenic,” “median nerve,” “ulnar nerve,” “brachial plexus,” “radial nerve,” “upper limb,” and “arm” to target specific anatomical locations. Procedural and injury-related terms such as “surgery,” “nerve injury,” “palsy,” and “paralysis” were also included to address the scope of surgical complications and their outcomes.

### Selection Criteria

The screening process began with titles, followed by abstracts, and concluded with full-text reviews to determine relevance. Only studies mentioning INIs of the upper limb were included, either in the title or as a major outcome of the study, with the full search strategy outlined in Table 1 . No restrictions were applied regarding publication date or article inclusion, and the search was conducted without exclusions beyond the scope of the topic, except that all selected manuscripts needed to be available as full texts in English and in peer-reviewed journals outside gray literature.

**Table 1:** Search string

Search string ( *n* = 534)
ALL = (Iatrogenic AND (Surgery AND (Median OR Ulnar OR (Brachial Plexus) OR Radial OR “Upper Limb” OR “Arm”) AND Nerve AND (Injury OR Palsy OR Paralysis)))

Note: Table 1 describes the search string used on Web of Science.

### Screening, Filtration, and Categorization

**Fig. 1:**
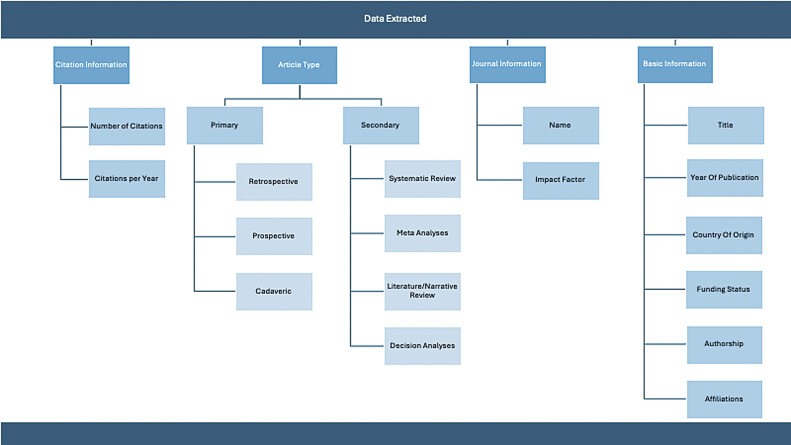
Data extracted.

Articles were then ranked based on the total number of citations across all databases. Three independent reviewers (S.S.G., A.G.K., H.S.) conducted separate searches, as per Lim et al. and Akmal et al. 3 12 Each reviewer compiled a list of the 50 most-cited articles, and any discrepancies between the lists were resolved through consensus. The final set of 50 publications underwent detailed analysis, both manually and using Web of Science metrics. 13 Fig. 1 outlines the data extracted for each study.

### Statistical Analysis

All the data were recorded and processed using Microsoft Excel (Microsoft, Washington, DC, United States). Shapiro–Wilk test confirmed nonparametric distribution; hence, median (± median absolute deviation) and 95% Bonnet Price confidence intervals were calculated. Percentages and confidence intervals (CIs) were rounded off to one decimal place. Tallies were tabulated and picturized as bar charts.

## Results

Table 2 presents the top 50 articles related to iatrogenic and operative nerve injuries, along with their citation counts and citations/year. These 50 articles collectively garnered a total of 2,576 citations, with individual citation counts ranging from 7 to 260, encompassing a total of 22703 live patients and 115 cadaveric subjects. The median citation count was 26.5 (± 15.0, 95% CI: 16–75), whereas the median citation rate was 3.7 citations per year (±1.7, 95% CI: 2.1–5.5), spanning a range of 0.7 to 12.1 citations per year.

**Table 2:** Top 50 cited articles

Title	Author (y)	Citations	Citations per year
Operative treatment of supracondylar fractures of the humerus in children—The consequences of pin placement 14	Skaggs et al (2001)	260	11.3
Lateral-entry pin fixation in the management of supracondylar fractures in children 52	Skaggs DL et al (2004)	196	9.8
A systematic review of medial and lateral entry pinning versus lateral entry pinning for supracondylar fractures of the humerus 29	Brauer CA et al (2007)	190	11.2
Nerve injuries associated with pediatric supracondylar humeral fractures: a meta-analysis 15	Babal JC et al (2010)	170	12.1
Traumatic and iatrogenic neurological complications after supracondylar humerus fractures in children 53	Brown IC et al (1995)	160	5.52
Ulnar nerve injury after K-wire fixation of supracondylar humerus fractures in children 54	Rasool MN et al (1998)	119	4.58
Plating osteosynthesis of mid-distal humeral shaft fractures: minimally invasive versus conventional open reduction technique 55	An ZQ et al (2010)	100	7.14
Complete transection of the median and radial nerves during arthroscopic release of post-traumatic elbow contracture 56	Haapaniemi T et al (1999)	96	3.84
Low incidence of ulnar nerve injury with crossed pin placement for pediatric supracondylar humerus fractures using a mini-open technique 57	Green DW et al (2005)	93	4.89
Iatrogenic radial nerve palsy after operative management of humeral shaft fractures 58	Wang JP et al (2009)	91	6.07
Factors associated with radial nerve palsy after operative treatment of diaphyseal humeral shaft fractures 59	Claessen FMAP et al (2015)	81	9
Iatrogenic ulnar nerve injury after the surgical treatment of displaced supracondylar fractures of the humerus: number needed to harm, a systematic review 60	Slobogean BL et al (2010)	77	5.5
Iatrogenic upper limb nerve injuries: a systematic review 18	Zhang J et al (2011)	75	5.77
Iatrogenic nerve injuries 6	Kretschmer T et al (2009)	53	3.53
Iatrogenic nerve injuries during shoulder surgery 61	Carofino BC et al (2013)	51	4.64
Results of crossed versus lateral entry K-wire fixation of displaced pediatric supracondylar humeral fractures: a systematic review and meta-analysis 62	Dekker, AE et al (2016)	50	6.25
Three lateral divergent or parallel pin fixations for the treatment of displaced supracondylar humerus fractures in children 63	Lee YH et al (2008)	48	3
Surgical interventions to treat humerus shaft fractures: a network meta-analysis of randomized controlled trials 64	Zhao JG et al (2017)	45	6.43
Iatrogenic brachial plexus injuries associated with open subpectoral biceps tenodesis a report of 4 cases 65	Rhee PC et al (2013)	44	4
The risks of Kirschner wire placement in the distal radius: a comparison of techniques 66	Hochwald NL et al (1997)	42	1.56
Treatment of humeral shaft fractures: minimally invasive plate osteosynthesis versus open reduction and internal fixation 67	Esmailiejah AA et al (2015)	41	4.56
The displaced supracondylar humerus fracture: indications for surgery and surgical options: a 2014 update 68	Ladenhauf HN et al (2014)	38	3.8
Minimally invasive plate osteosynthesis vs conventional fixation techniques for surgically treated humeral shaft fractures: a meta-analysis 69	Hu XQ et al (2016)	38	4.75
Ultrasonographic evaluation of the iatrogenic peripheral nerve injuries in upper extremity 9	Karabay N et al (2010)	36	2.57
Iatrogenic ulnar nerve injury after pin fixation and after antegrade nailing of supracondylar humeral fractures in children 70	Eberl R et al (2011)	27	2.08
Comparison of lateral entry with crossed entry pinning for pediatric supracondylar humeral fractures: a meta-analysis 71	Na YY et al (2018)	26	4.33
The safe zone for avoiding suprascapular nerve injury in bone block procedures for shoulder instability. A cadaveric study 72	Longo UG et al (2015)	25	2.78
Treatment of non-union of humerus diaphyseal fractures: a prospective study comparing interlocking nail and locking compression plate 73	Singh AK et al (2014)	23	2.3
Iatrogenic nerve palsy occurs with anterior and posterior approaches for humeral shaft fixation 74	Streufert BD et al (2020)	22	5.5
Iatrogenic ulnar neuropathies post-pinning of displaced supracondylar humerus fractures in children 75	Rose REC et al (2002)	22	1
The risk of injury to neurovascular structures from distal locking screws of the Unreamed Humeral Nail (UHN): a cadaveric study 76	Noger M et al (2007)	22	1.29
Case-match controlled comparison of minimally invasive plate osteosynthesis and intramedullary nailing for the stabilization of humeral shaft fractures 77	Davies G et al (2016)	21	2.63
Iatrogenic posterior interosseous nerve injury - is transosseous static locked nailing of the radius feasible 78	Tabor, OB et al (1995)	20	0.690
Iatrogenic ulnar nerve injury after percutaneous cross-pinning of supracondylar fracture in a child 79	Taniguchi Y et al (2000)	20	0.833
Treatment of fifth metacarpal neck fractures with antegrade single elastic intramedullary nailing 80	She YS et al (2017)	19	2.71
Iatrogenic radial nerve palsy after humeral shaft nonunion repair: more common than you think 81	Kakazu R et al (2016)	17	2.13
Time from injury to surgical fixation of diaphyseal humerus fractures is not associated with an increased risk of iatrogenic radial nerve palsy 82	Shoji K et al (2017)	16	2.29
Radial nerve injury during double plating of a displaced intercondylar fracture 83	Lim R et al (2012)	16	1.33
Iatrogenic radial nerve injury with cannulated fixation of medial epicondyle fractures in the pediatric humerus: a report of 2 cases 84	Marcu DM et al (2011)	16	1.23
Medial and lateral crossed pinning versus lateral pinning for supracondylar fractures of the humerus in children: decision analysis 85	Lee KM et al (2012)	16	1.33
Comparing iatrogenic radial nerve lesions in humeral shaft fractures treated with helical or straight philos plates: a 10-year retrospective cohort study of 62 cases 86	Da Silva T et al (2020)	14	3.5
What is the real rate of radial nerve injury after humeral nonunion surgery? 87	Koh J et al (2020)	12	3
A mini-open approach to medial pinning in pediatric supracondylar humeral fractures may be safer than previously thought 88	Rees AB et al (2022)	12	6
Comparison of lateral entry and crossed entry pinning for pediatric supracondylar humeral fractures: a meta-analysis of randomized controlled trials 89	Zhao H et al (2021)	12	4
Crossed versus lateral K-wire fixation of supracondylar fractures of the humerus in children: a meta-analysis of randomized controlled trials 90	Carrazzone OL et al (2021)	12	4
Paediatric supracondylar humeral fractures: a technique for safe medial pin passage with zero incidence of iatrogenic ulnar nerve injury 91	Woo CY et al (2018)	11	1.83
Lateral versus cross pinning in paediatric supracondylar humerus fractures: a meta-analysis of randomized control trials 92	Kwok SM et al (2021)	10	3.33
Iatrogenic injuries of the palmar branch of the median nerve following volar plate fixation of the distal radius 17	Samson D et al (2017)	8	1.14
Iatrogenic peripheral nerve injuries-Common causes and treatment: a retrospective single-center cohort study 93	Hara T et al (2021)	7	2.3
Postoperative ulnar neuropathy is not necessarily iatrogenic: a prospective study on dynamic ulnar nerve dislocation at the elbow 94	Billmann FG et al (2014)	7	0.7

The most cited peer-reviewed publication was “Operative Treatment of Supracondylar Fractures of the Humerus in Children—The Consequences of Pin Placement” by Skaggs DL et al. (2001), published in Journal of Bone and Joint Surgery - American Volume (JBJS), which accumulated 260 citations. 14 However, when ranked by citations per year, the most impactful publication was “Nerve Injuries Associated with Pediatric Supracondylar Humeral Fractures: A Meta-analysis” by Babal JC et al., published in Journal of Pediatric Orthopaedics with a median of 10 (±3, 95% CI: 18, 18) citations/year and provided a comprehensive analysis of surgical approaches for nerve injuries in children, emphasizing the risks of iatrogenic nerve damage in the upper limb. 15

The studies analyzed were published in journals with a median impact factor (IF) of 2 (±0.6, 95% CI: 1.5, 2.9). The Journal of Pediatric Orthopaedics published the most studies within the top 50 cited articles on INIs, with 8 publications (IF: 1.4), followed by the Journal of Orthopaedic Trauma with 7 publications (IF: 1.6), and the Journal of Bone and Joint Surgery—American Volume with 4 publications (IF: 4.4). The highest IF journals included Journal of Bone and Joint Surgery—American Volume (4 publications, IF: 4.4), Arthroscopy (1 publication, IF: 4.4), and American Journal of Sports Medicine (1 publication, IF: 4.2).

**Fig. 2:**
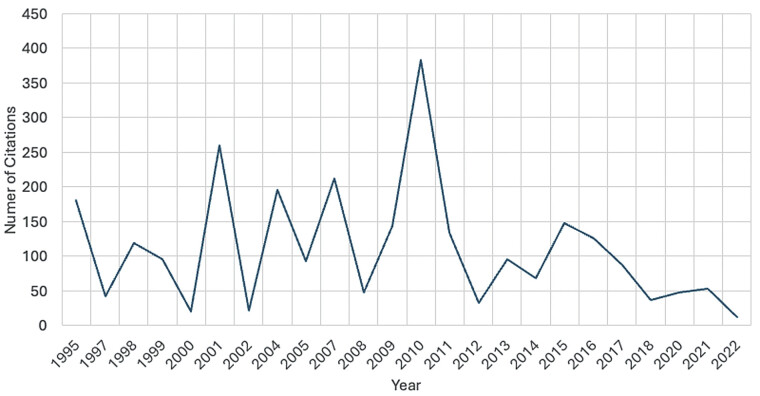
The number of citations of articles published, of the those within the top 50 cited articles, depicted by year.

Figs. 2^3^,4,5,6 and Tables 1 2 3 4 5 6 7 8 provide a comprehensive summary of our findings, emphasizing key temporal trends and thematic insights.

**Table 3:** Authors with more than 1 entry in the top 50 cited articles

Name	Institution	First author	Last author	Other author	Total
Skaggs, DL	Children's Hospital, Los Angeles, California, United States	2	0	0	2
Kay, RM	Children's Hospital, Los Angeles, California, United States	0	1	1	2
Rasool, MN	King Edward VIII Hospital, Durban, South Africa	1	1	0	2
Spinner, Robert J	Mayo Clinic, Rochester, Minnesota, United States	0	0	2	2
Bishop, Allen T	Mayo Clinic, Rochester, Minnesota, United States	0	0	2	2
Shin, Alexander Y	Mayo Clinic, Rochester, Minnesota, United States	0	2	0	2
Mir, Hassan R	University of South Florida, Florida, United States	0	2	0	2

Note: Of the 259 distinct contributing authors, a total of 7 authors contributed more than 1 article to this study.

**Table 4:** Study themes

Theme	Number
Supracondylar fractures of the humerus	21
Humeral shaft fracture	10
Other	5
Diaphyseal humeral fractures	4
Shoulder surgery	4
Surgical techniques	2
Supracondylar humerus fractures	2
Distal radius fracture	2
Arthroscopic release of posttraumatic elbow contracture	1
Bicep tenodesis	1
Metacarpal neck fracture	1
Intercondylar humerus fracture	1
Medial epicondyle fractures of the humerus	1
Humeral nonunion	1
Ulnar nerve dislocation	1

**Table 5:** Nerves referenced

Nerve	Number
Ulnar nerve	23
Multiple nerves	18
Radial nerve	14
Brachial plexus	1
Suprascapular nerve	1
Posterior interosseous nerve	1
Median nerve	1

**Table 6:** Institutions associated with more than 1 article

Institutions	Number
Mayo Clinic, Rochester, United States	3
Children's Hospital, Los Angeles, United States	2
BC Children's Hospital, University of British Columbia, Vancouver, Canada	2
Seoul National University College of Medicine, Seoul, South Korea	2
Tianjin Medical University, Tianjin, China	2
Tianjin Hospital, Tianjin, China	2
Ningbo No. 6 Hospital, Ningbo, Zhejiang, China	2
Shangyu People's Hospital of Shaoxing, Zhejiang, China	2
Ninghai Hospital of Traditional Chinese Medicine, Ninghai, Zhejiang, China	2
University of South Florida, Florida, United States	2

**Table 7:** Country of origin

Country	Number
United States	16
China	6
Canada	3
Germany	2
Austria	2
Singapore	2
Japan	2
Australia	2
Republic of Korea	2
Taiwan	1
New Zealand	1
Netherlands	1
South Africa	1
Iran	1
Turkey	1
Italy	1
India	1
Jamaica	1
Switzerland	1
UK	1
Sweden	1
Brazil	1

**Table 8:** Articles published by each journal

Source title	Count	IF
Journal of Pediatric Orthopaedics	8	1.4
Journal of Orthopaedic Trauma	7	1.6
Journal of Bone and Joint Surgery-American Volume	4	4.4
Journal of Shoulder and Elbow Surgery	3	2.9
Journal of Orthopaedic Surgery And Research	3	2.8
ANZ Journal of Surgery	2	1.5
Injury-International Journal of the Care of the Injured	2	2.2
Archives of Orthopaedic and Trauma Surgery	2	2
Journal of Hand Surgery-American Volume	2	2.1
Acta Orthopaedica	1	2.5
Journal of Orthopaedic Science	1	1.5
Journal of Hand Surgery-Asian-Pacific Volume	1	0.5
Singapore Medical Journal	1	1.7
BMC Musculoskeletal Disorders	1	2.2
West Indian Medical Journal	1	0.2
Knee Surgery Sports Traumatology Arthroscopy	1	3.3
Trauma Monthly	1	0.2
European Journal of Radiology	1	3.2
Current Opinion in Pediatrics	1	2.2
American Journal of Sports Medicine	1	4.2
PLoS One	1	2.9
Neurosurgery Clinics of North America	1	2
Journal of Trauma-Injury Infection and Critical Care	1	3.0
Arthroscopy - The Journal of Arthroscopic and Related Surgery (Arthroscopy)	1	4.4
International Orthopaedics	1	2
World Journal of Surgery	1	2.3

**Fig. 3:**
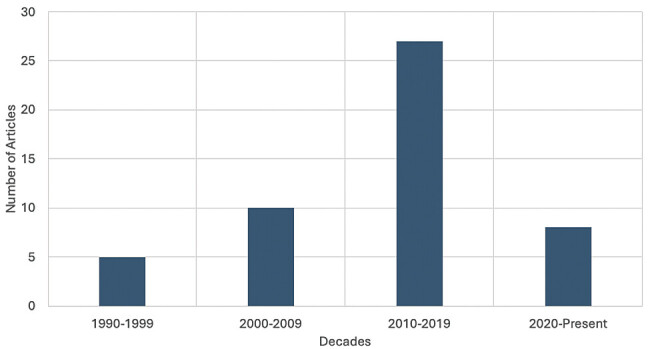
Articles included in the top 50 citations published per decade.

**Fig. 4:**
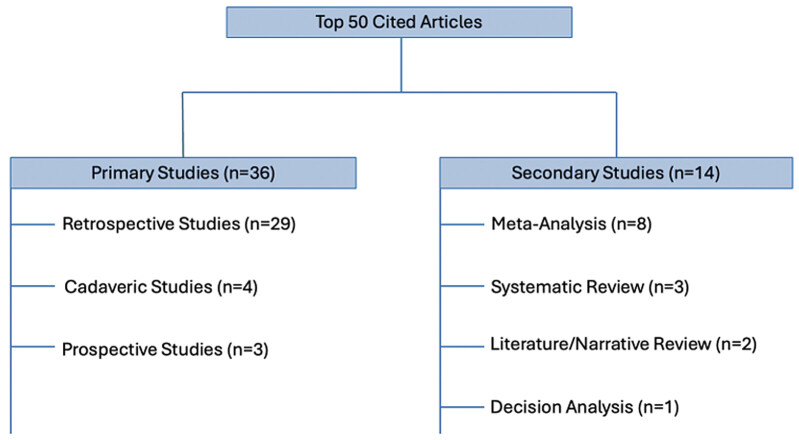
Types of study.

**Fig. 5:**
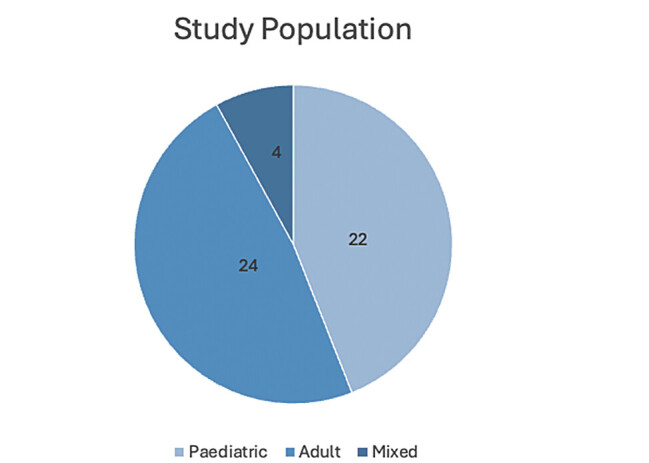
Study population.

**Fig. 6:**
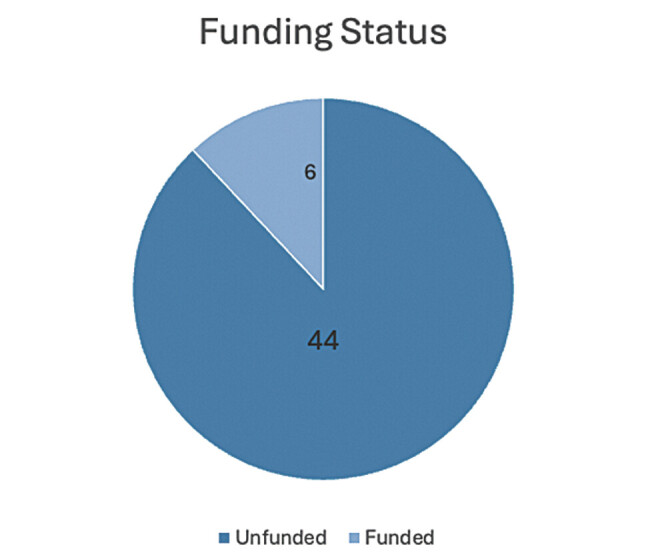
Funding status.

## Discussion

### Principal Findings

The top 50 articles were published over a span of 27 years (1995–2022), with the highest citation counts recorded in 2007 (383 citations) and 2010 (212 citations). Over half of the studies (54%, *n* = 27) were published during the 2010s. Notably, seven authors (2.7%, 7/259) and 10 institutions (13.3%, 10/75) contributed more than one article. The most frequently discussed themes included supracondylar fractures of the humerus ( *n* = 21) and humeral shaft fractures ( *n* = 10). Among nerve injuries, the ulnar nerve was the most commonly addressed ( *n* = 23), followed by injuries involving multiple nerves ( *n* = 18) and the radial nerve ( *n* = 14).

### Peak Trends in Research Output

Despite upper limb nerve injury being iatrogenic in 17% of cases, 8 the first highly cited study was not conducted until 1995. The timeframe that yielded the top cited articles was between years 1995 to 2022. This finding aligns with bibliometric trends in related fields such as wrist surgery where the earliest paper dates back to 1991 and most studies were published between 2000 and 2010. 16 This may be due to the anatomical and clinical relationship between wrist fractures and nerve injuries, as concomitant injures are common. 17

The peak research activity was in the 2010s, with this decade experiencing nearly a 3-fold increase compared with the prior decade, reflecting a growing awareness of INI as a significant clinical burden of disease, likely driven by advances in surgical techniques, patient outcomes, and medicolegal considerations. 7 9 18 19 The limited highly cited literature before 2010 shows that research on iatrogenic upper limb nerve injuries is recent and increasingly recognized as an avoidable perioperative complication. In addition, global efforts like the World Health Organization's Safe Surgery Saves Lives campaign placed a spotlight on avoiding preoperative surgical complications, including INIs, by emphasizing nerve monitoring and surgical safety protocols with extra emphasis on preoperative planning. 20 Therefore, leading to a greater focus on quantifying the effects and consequences of such injuries.

### Appropriate Management of Iatrogenic Peripheral Nerve Injuries

Similarly, the advent of the getting it right first time initiative dovetails with this concept, aiming to reduce future complications and improve the landscape of care within the NHS. 21 22 Within the timeframe, more emphasis was put on outcomes-based research, aiming to understand and improve functional outcomes postoperatively, potentially sparking more impactful studies on INIs. 23 However, the delay in referral of INI to a regional specialist center still remains a significant issue. 24 Therefore, particular efforts should be made to prioritize regional and center-based quality improvement projects to enhance outcomes and streamline referrals. Adopting a model like that used for esophagogastric cancers, with centralized regional tertiary centres for complex cases, could improve outcomes for INI patients while reducing regional disparities and health inequalities. 25 26 27

### Optimal Metrics for Citations

We used citation count and citations per year as a measure for impact on clinical practice of articles included in the analysis. Both metrics have their advantages with citation count highlighting articles that have had a long-lasting influence in the field, regardless of publication date as well as identifying foundational or landmark studies. Yet, the Matthew Effect where highly cited articles tend to attract more citations simply because they are already visible may augment and perpetuate an increase in the number of citations than older and lesser cited articles. 28 The studies with the highest number of total citations, and highest citations/year, are based around pediatric supracondylar humeral fractures, suggesting that such fractures are a key area of research interest, potentially due to its prevalence, clinical implications, and potential for complications. 15 29

### Concentration of Cited Peer-Reviewed Journals

Similarly, considering the niche nature of iatrogenic upper limb nerve injuries as a topic, often limited to pediatric orthopaedics, our finding of a median IF of 2 appears appropriate. This is further supported by the distribution of articles across the 26 journals analyzed, with five journals contributing three or more articles each. This pattern aligns well with Bradford's Law, which highlights how a small number of journals often dominate the publication landscape within specialized fields. 30 As such, the concentration of research within these key journals not only underscores their role as authoritative sources in the field but also facilitates the dissemination of high-quality evidence, shaping clinical guidelines and influencing standardized practices. This trend highlights the importance of targeting these journals for future research contributions to maximize their clinical impact and ensure that advancements in understanding and management of iatrogenic upper limb nerve injuries are effectively translated into improved patient outcomes and care strategies.

### Role of National Funding within Research Impact

The United States contributed 32% of cited articles, reflecting its leadership in orthopaedic surgery and research, supported by significant National Institutes of Health (NIH) funding. 31 32 Institutions like Washington University, ranked #1 in NIH orthopaedic funding since 2009, and the Mayo Clinic, with $1.14 billion in research funding in 2023, drive globally impactful research through substantial resources and high patient volumes. 33 34

Asia accounted for 32% of cited articles in this analysis, surpassing Europe by 14%, reflecting its significant growth in research output over recent decades, driven by substantial investments in research and development. China's six publications highlight its remarkable progress in orthopaedic research, leveraging high surgical volumes to study complications like INIs. 35 The rising contributions from institutions like the Mayo Clinic and emerging Asian leaders reflect a global shift in research excellence. This trend emphasizes the potential for cross-regional collaboration to advance clinical practice, improve surgical outcomes, and address complications more effectively.

### Trends in Specific Named Nerves Involved in Iatrogenic Injury

The ulnar nerve was the most frequently reported iatrogenic injury, appearing in 46% of the studies. This is likely due to several factors, particularly its anatomical vulnerability and superficial plane, making it highly prone to compression and direct trauma during surgical procedures. 36 37 Additionally, common surgeries such as open reduction and internal fixation for distal humerus fractures in trauma, and either traumatic or elective partial or total elbow arthroplasty place the ulnar nerve at significant risk due to its proximity to the surgical field. 38 Given that the course of the ulnar nerve can vary substantially between individuals, precise identification and safeguarding of the nerve can be challenging during complex elbow procedures, increasing the risk of iatrogenic injury. 39 40 Moreover, scar tissue or altered anatomy in revision surgeries may obscure the normal landmarks of the ulnar nerve, leading to inadvertent entrapment within sutures or hardware. 41 42 43 44 Finally, prolonged operative times, coupled with potential fluctuations in local blood supply, can further compromise the nerve's integrity, thereby elevating its risk of perioperative injury. 45 46 47

In contrast, the median and posterior interosseous nerves were the least frequently reported, each mentioned in only one study. This is likely attributed to their deeper anatomical positioning, which offers increased protection from surgical trauma as well as protective measures taken to avoid their encounter in widely accepted surgical approaches. 48 Given the findings outlined by Majeed et al., that neurological injuries accounted for 24.5% of damages paid due to patient dissatisfaction between 2008/09 and 2018/19 in the NHS, the need to address iatrogenic nerve complications is evident. 49 Mitigating ulnar nerve injuries through meticulous surgical techniques, enhanced preoperative planning, being familiar with its course and appropriate handling as well as the adoption of nerve-sparing approaches are crucial to improving patient outcomes and reducing medicolegal liabilities.

### Limitations

Relying solely on the Web of Science database may have resulted in the exclusion of relevant articles indexed in other platforms like PubMed, Scopus, or Embase, as these individual databases lack citation data, making them unsuitable for conducting a bibliometric analysis. 50 To combat this, all included studies from the Web of Science database were cross-verified and identified at least one of these databases. While our search encompassed all INIs of the upper limb, the majority of our analysis focused on sensorimotor nerves, not focusing on sensory nerves such as the cutaneous nerves. Although keywords and MeSH terms were pertinently selected, there is a risk of missing relevant studies due to potential iterations in terminology or incomplete indexing in the database. Nevertheless, broad terms were utilized to capture the most relevant literature in our final analysis and to ensure appropriate reproducibility. In addition, citation counts do not necessarily reflect the quality, influence, or clinical relevance of the literature but may act as a proxy to reflect influence, impact, and applicability.

### Future Work

Future studies could focus on assessing the contemporary impact of supracondylar humeral fractures on patients and health care systems. Comparing these findings with historical data could provide insights into temporal trends and highlight factors influencing the evolution of fracture management. Advances such as minimally invasive techniques, refined pinning methods, and nerve-sparing approaches have significantly reshaped treatment protocols, and their specific contributions to patient outcomes and system-level changes warrant detailed exploration. 51 Furthermore, these reviews could yield landmark recommendations, shaping management guidelines, surgical decision-making, and standard-of-care practices, thereby reflecting the growing prominence and acceptance of these advancements.

## Conclusion

This bibliometric analysis highlights the most-cited articles on iatrogenic upper limb nerve injuries, showcasing influential research, key trends, and global contributions. The prominence of institutions like the Mayo Clinic and growing output from Asia reflects a global shift in research leadership, emphasizing opportunities for collaboration to refine surgical techniques and enhance patient care. Our findings underscore the need for targeted preventive strategies, particularly for ulnar nerve injuries, which are most frequently reported due to their anatomical vulnerability and risk during common procedures. By identifying landmark studies and trends, this analysis provides valuable insights to guide future research and improve outcomes for patients with INIs.
